# Comparative evaluation of post-operative pain and tissue response in patients undergoing conventional surgical approaches with or without diode laser: A randomised clinical study

**DOI:** 10.6026/973206300214762

**Published:** 2025-12-15

**Authors:** Khushbu Jain, Dennis V Abraham, Sachin Malagi, Rangoli Bhirad, Shweta Mishra, Amit Mihani

**Affiliations:** 1 Department of Periodontics and Implantology, Maitri College of Dentistry and Research Institute, Anjora, Durg, Chhattisgarh, India; 2 Department of Periodontics, Maitri College of Dentistry and Research Centre, Anjora, Durg, Chhattisgarh, India; 3 Department of Periodontics, Kalka Dental College Meerut, Uttar Pradesh, India

**Keywords:** Diode laser, Kirkland flap, modified widman flap, periodontitis, split-mouth

## Abstract

Periodontal regeneration often requires surgical intervention, yet conventional procedures may lead to postoperative discomfort and inflammation, raising interest in diode laser-assisted therapy. Therefore, it is of interest to compare healing outcomes after Kirkland and Modified Widman flap surgeries performed with or without 976 nm diode laser exposures in 80 sites. Laser-treated groups showed greater reductions in probing depth and gingival inflammation, with Group B exhibiting the highest SBI reduction and Group D demonstrating the greatest CAL gain. Pain scores favored diode laser use, particularly in Group D on day 1. Diode laser adjuncts significantly enhanced clinical healing parameters and reduced discomfort, supporting their beneficial role in periodontal surgery.

## Background:

Periodontal therapy, nowadays does not only involve in arresting the disease process, but also in the regeneration of the tissues that have been lost during the process [1]. The ultimate goal of periodontal therapy is to regenerate the supporting tissues lost as a consequence of inflammatory periodontal disease. This implies the formation of a new connective tissue attachment, i.e. new cementum with inserting collagen fibers at previously diseased root surfaces and also the re-growth of alveolar bone. Mechanical debridement is an effective treatment approach for periodontal disease [2]. Due to limited access to areas like furcations, concavities, and developmental grooves, complete removal of bacterial deposits and their toxins from the root surface is not achievable with non-surgical mechanical therapy; therefore, surgical intervention is required [3]. The aim of the surgical procedure involving treatment of periodontal pockets by Modified Widman Flap (MWF) is at re-attachment and re-adaptation of the pocket walls rather than the surgical eradication of the outer walls of the pockets [4]. Though complete mechanical debridement is the "gold standard" of periodontal treatment, [5] it still does not eliminate the micro-organisms in the soft tissue wall of the pocket, nor is complete resection of the diseased tissues possible. In recent years, various innovations in adjunctive treatments have been introduced to enhance the clinical effectiveness of scaling and root debridement. Amongst the various new technologies offered, therapeutic laser treatment, also referred to as laser bio stimulation, offers numerous benefits. Primarily being nonsurgical, it promotes tissue healing, reduces edema, inflammation and pain [6]. Lasers aid in the shaping of gingiva and prevent epithelium from penetrating the margins of gingiva. There is a suggestion that lasers modify the behaviour of cells by influencing membrane calcium channels or the mitochondrial respiratory chain. This leads to an increase in angiogenesis, collagen synthesis, and growth factor release, all of which speed up the healing process of wounds [7]. It has also been found to promote fibroblast maturation and proliferation, macrophage phagocytosis, and lymphocyte activation, thereby aiding in the formation of a new connective tissue attachment [8, 9- 10]. Therefore, it is of interest to compare evaluation of post-operative pain and tissue response in patients undergoing conventional surgical approaches with or without diode laser.

## Materials and Methods:

20 patients (9 males 11 females), who reported to the out-patient Department of Periodontology at Maitri college of dentistry and research centre, Anjora, Durg, Chhattisgarh were included in the study.

## Inclusion and exclusion criteria:

## Inclusion criteria:

[1] Systemically healthy subjects.

[2] Subjects who maintain good oral hygiene.

[3] Subjects with probing pocket depth between 5-7mm.

[4] Subjects who have not taken antibiotics within 6 months of initial examination and who do not require antibiotic premedication for any systemic condition.

[5] Subjects who have not undergone any kind of periodontal therapy in the past 6 months.

[6] Subjects with all the posterior teeth present.

## Exclusion criteria:

[1] Pregnant or lactating women.

[2] Subjects with substance abuse.

[3] Subjects with history of drug allergy

[4] Subjects with intrabony defects

[5] Teeth with grade II or III mobility

## Study design:

The present study is a single blinded, split mouth randomised clinical trial executed from May 2024 - December 2025. Ethical and scientific clearance was obtained from the MCDRC institutional ethical committee and Review Board of the Maitri college of dentistry and research institute with ref no: MCDRC/2024/APR/433 and MCDRC/2024/APR/432 respectively. A total of 20 subjects with 80 sites were selected, following Phase-I therapy. After 4 weeks of oral hygiene reinforcement, subjects were recalled for clinical evaluation at baseline and 6 months' post-operative. Sulcus bleeding index (SBI), probing pocket depth (PPD), clinical attachment level (CAL) and gingival inflammatory index (GI) were recorded. Radiographs (OPG) were taken to analyse the type of bone loss. Pain assessment was evaluated using visual analogue numeric pain scale (VAS) on 1st and 3rd day postoperatively.

## Initial therapy (Pre-surgical assessments):

Full mouth scaling and root planing was performed. Oral hygiene instructions were given and subjects were advised to use 10ml 0.2% chlorhexidine gluconate mouth wash twice daily for 14 days. At the end of 2 weeks, patients were revaluated and if they qualified for entry to the study then baseline examination was carried out which included the recording of following parameters:

Gingival Index - GI Loe H and Silness P in 1963, Sulcus Bleeding Index -SBI (Muhlemann and sons 1970), Probing Pocket Depth (PPD), Clinical Attachment Level (CAL).

## Surgical procedure:

Intra-oral and extra oral asepsis was achieved with povidone iodine solution, prior to administration of local anaesthetic solution (2% lignocaine hydrochloride with 1:2,00,000 adrenaline) to the subjects and all the subjective symptoms were evaluated for anaesthesia. Standardized surgical procedures for test and control sites were performed. The study consisted of four groups, each comprising 20 sites. Group A underwent Kirkland flap surgery without diode laser exposure, while Group B received the same procedure with adjunctive diode laser use. Group C was treated using the Modified Widman flap technique without laser application, whereas Group D underwent the Modified Widman flap with diode laser exposure. This design allowed direct comparison of conventional and laser-assisted periodontal surgeries across both flap techniques. Only the subjects in group B and D were treated with Diode Laser irradiation, an indium gallium and arsenide diode laser (WOODPECKER) with a wavelength 976nm and a power of 1 watt was irradiated on inner surface of the flap, exposed bone and exposed root structures involved in continuous non-contact mode for 30 seconds before flap closure. The time interval between the surgeries was 1 week. Periodontal dressing was not given in any of the patient, so as to analyse the gingival inflammation after 3rd and 7th day respectively. All the necessary post-operative instructions were given to the patient and regimen was followed. Post-operative instructions were given, including avoiding brushing at the surgical site for one week. Subjects received antibiotics (Amoxicillin 500 mg + Clavulanic acid 125 mg) and analgesics (Aceclofenac 100 mg + Paracetamol 325 mg) twice daily for 3 days, along with saline rinses for a week. Follow-ups were scheduled on days 1 and 3 to assess healing. Sutures were removed on day 7 after betadine irrigation. Clinical parameters were re-evaluated at 6 months. After the completion of surgery, subjects were asked to evaluate pain at 1st and 3rd day as per Visual Analogue Scale (VAS). The treatment protocol consisted of seven sequential visits. During the first visit, a detailed case history was recorded and written informed consent was obtained. The second visit included oral prophylaxis and alginate impression recording, followed by baseline clinical measurements of PPD, CAL, and SBI at the third visit. At the fourth visit, subjects were randomized, and periodontal surgery was performed in the respective quadrants with or without diode laser exposure. Postoperative discomfort was assessed using the VAS scale during the fifth visit, and both VAS and gingival inflammation (GI) were evaluated at the sixth visit. Finally, at the seventh visit, conducted six months postoperatively, probing pocket depth, clinical attachment level, and sulcus bleeding index were reassessed to determine treatment outcomes.

## Results:

The study involved 80 subjects divided equally into four groups (A, B, C, and D; 20 in each group) to evaluate clinical outcomes over six months. The parameters assessed included Probing Pocket Depth (PPD), Clinical Attachment Level (CAL), Visual Analog Scale (VAS), Sulcular Bleeding Index (SBI), and Gingival Inflammatory Index (GI). A significant reduction in PPD was observed across all groups from baseline to six months. Group B showed the highest reduction (mean drop from 6.2 mm to 4.14 mm), followed by Group C, A, and D. Intragroup and intergroup comparisons revealed statistical significance (p < 0.05). Group D and B showed the greatest improvement in CAL from baseline to six months (mean reduction of ~3.8 mm), while Groups A and C followed. Group A showed the least gain. The differences were statistically significant, especially between Group A and Groups B/D. Pain levels declined significantly from baseline to Day 3 across all groups. On Day 1, Group D reported the most relief, while Group C reported the least. By Day 3, Group C showed the lowest VAS score (mean 1.77), indicating better pain management over time. Intergroup comparisons confirmed significance. SBI scores reduced notably from baseline to six months, with Group B showing the most reduction (mean drop from 2.54 to 0.68), followed by Group D, C, and A. Differences were statistically significant. From baseline to Day 7, the GI scores decreased significantly. Group D showed the greatest reduction from baseline to Day 7 (3.29 to 1.17), followed by Groups C, A, and B. The third to seventh day comparison showed the highest improvement in Group C. Most intergroup differences were not statistically significant at Day 7 (Table 1 - see PDF).

## Discussion:

This study assessed the adjunctive role of diode lasers (DL) in periodontal flap surgeries, emphasizing postoperative healing, clinical outcomes, and patient comfort. Periodontal therapy primarily aims to halt disease progression, promote regeneration of supporting tissues, and sustain long-term periodontal stability. Conventional flap techniques, including the Kirkland and Modified Widman approaches, are well established for achieving these therapeutic objectives [11]. Although lasers have been introduced to enhance periodontal outcomes, existing evidence remains variable, with some reports indicating limited additional benefits. Diode lasers operating within 800-980 nm continue to be widely utilized due to their cost-effectiveness, portability, and strong absorption in hemoglobin and pigmented tissues, enabling efficient bactericidal activity, hemostasis, and minimal thermal damage to adjacent hard tissues [12]. In this study, their application during surgical procedures demonstrated effective soft-tissue management with minimal adverse effects. Pain evaluation, a key postoperative parameter, was measured using the Visual Analog Scale (VAS). Laser-treated sites exhibited significantly lower pain scores on Days 1 and 3. This may be attributed to the laser's ability to modulate neural conduction, decrease inflammatory mediators, and promote endogenous analgesic release. These findings align with earlier research demonstrating reduced postoperative discomfort with DL use [13, 9, 14]. However, other studies reported no significant differences in pain reduction [15, 16], suggesting that outcomes may vary depending on energy settings, technique, and individual tissue responses. Gingival inflammation showed improvement by Day 7 across all groups, though statistical significance was not observed. The greatest reduction occurred in the Modified Widman flap group, consistent with earlier observations [13]. Conversely, some studies have reported notable reductions in inflammation following laser-assisted therapy [10], indicating that effectiveness may depend on specific laser parameters and dosimetry. The biological effects may be partially explained through the Arndt-Schultz law [17], which states that low doses stimulate cellular activity, moderate doses inhibit, and high doses may be damaging. DLs have also been associated with increased ATP production, enhanced microcirculation, improved leukocyte function, and upregulated antioxidant mechanisms, all contributing to better healing.

Clinical Attachment Level (CAL), a critical indicator of periodontal stability [18], improved significantly in all groups after 6 months. The greatest gain was observed in the Modified Widman flap combined with DL, followed by the Kirkland flap with DL. These findings support the adjunctive benefits of diode lasers in promoting periodontal regeneration [19, 20, 18]. Probing Pocket Depth (PPD) also reduced significantly across all groups, with the most substantial reduction noted in the Kirkland flap with DL. Comparable results have been documented in earlier research [10, 21, 22], although some studies reported similar improvements in both laser-treated and conventional groups [23]. Sulcus Bleeding Index (SBI) demonstrated significant reduction in all groups, with the maximum improvement occurring in the Kirkland flap with DL. This aligns with previous observations [22], although other studies found no clear advantage of laser use for bleeding control [24, 25]. Despite these variations, the overall outcomes of the present study indicate that diode laser-assisted flap surgeries offer distinct advantages in reducing postoperative pain, improving soft-tissue healing, and lowering pocket depth. Laser use in dentistry continues to be regarded as safe, with no evidence of carcinogenic effects. Nonetheless, adherence to established safety protocols, especially eye protection, remains essential during clinical application [26]. With standardized parameters and appropriate technique, diode lasers present a valuable adjunct to conventional periodontal therapy, contributing to enhance healing and improved clinical outcomes.

## Conclusion:

Despite available data on laser use in periodontics, few long-term trials exist, leading to ongoing debate and limited acceptance of low-level lasers in routine dental therapy. In this study, diode laser use significantly reduced post-operative pain and improved patient comfort. Results align with previous clinical and histological findings. However, limitations include a small sample size and lack of microbial assessment, making generalization difficult.
